# High-fat diet induces cardiac remodelling and dysfunction: assessment of the role played by SIRT3 loss

**DOI:** 10.1111/jcmm.12556

**Published:** 2015-03-17

**Authors:** Heng Zeng, Venkata Ramana Vaka, Xiaochen He, George W Booz, Jian-Xiong Chen

**Affiliations:** Department of Pharmacology and Toxicology, The University of Mississippi Medical Center, School of MedicineJackson, MS, USA

**Keywords:** Sirtuin 3, HIF-1α, HIF-2α, High-fat diet, reactive oxygen species, cardiac function

## Abstract

Mitochondrial dysfunction plays an important role in obesity-induced cardiac impairment. SIRT3 is a mitochondrial protein associated with increased human life span and metabolism. This study investigated the functional role of SIRT3 in obesity-induced cardiac dysfunction. Wild-type (WT) and SIRT3 knockout (KO) mice were fed a normal diet (ND) or high-fat diet (HFD) for 16 weeks. Body weight, fasting glucose levels, reactive oxygen species (ROS) levels, myocardial capillary density, cardiac function and expression of hypoxia-inducible factor (HIF)-1α/-2α were assessed. HFD resulted in a significant reduction in SIRT3 expression in the heart. Both HFD and SIRT3 KO mice showed increased ROS formation, impaired HIF signalling and reduced capillary density in the heart. HFD induced cardiac hypertrophy and impaired cardiac function. SIRT3 KO mice fed HFD showed greater ROS production and a further reduction in cardiac function compared to SIRT3 KO mice on ND. Thus, the adverse effects of HFD on cardiac function were not attributable to SIRT3 loss alone. However, HFD did not further reduce capillary density in SIRT3 KO hearts, implicating SIRT3 loss in HFD-induced capillary rarefaction. Our study demonstrates the importance of SIRT3 in preserving heart function and capillary density in the setting of obesity. Thus, SIRT3 may be a potential therapeutic target for obesity-induced heart failure.

## Introduction

Obesity is prevalent in the Western and developing worlds. According to The International Union of Nutritional Sciences, obesity rates could be as high as 45–50% in United States by the year 2025 1. Obesity is considered an independent risk factor for heart failure 2. Mortality because of cardiovascular diseases such as stroke, coronary heart disease, congestive heart failure and cardiomyopathy are strongly associated with obesity 3. Studies have shown the important role of obesity in cardiac dysfunction, LV hypertrophy and dilatation 4,5; however, the mechanisms by which obesity causes adverse remodelling and impairs performance of the heart are poorly understood.

Obesity has been linked to a Western diet high in fat. The heart exhibits a high rate of fatty acid oxidation to meet the tremendous need for adenosine triphosphate (ATP) for contraction. In fact, mitochondria comprise ≈30% of cardiac myocytes by volume 6. Consequently, mitochondrial dysfunction negatively impacts on contractile performance of the heart. Recently, we reported that cardiomyopathy observed with another metabolic disorder, obese diabetes was associated with increased reactive oxygen species (ROS) formation in the heart because of a reduction in SIRT3 expression 7. The NAD^+^-dependent protein deacetylase SIRT3 is important for mitochondrial function, in part by regulating redox balance and anti-oxidant defenses 8. More recently, SIRT3 has gained attention in its particular roles in metabolic syndrome. SIRT3 is highly expressed in metabolic tissues such as liver and skeletal muscle. We also, observed that loss of SIRT3 in diabetic hearts was associated with capillary rarefaction 7. Here, we tested the hypothesis that high-fat diet (HFD) impairs cardiac performance by a mechanism involving impairment of SIRT3 signalling in the heart. Wild-type (WT) and SIRT3 knockout (SIRT3 KO) mice were fed with HFD for 16 weeks to develop a diet-induce obesity (DIO) model. Using this DIO mouse model, we have examined the effects of SIRT3 deficiency on the HFD-induced cardiac dysfunction. Moreover, we have explored the potential mechanisms by which SIRT3 deficiency regulates HFD-induced loss of capillaries.

## Materials and methods

### Materials

The antibody for SIRT3 (5490) was purchased from Cell Signalling Technology (Danvers, MA, USA). Bovine serum albumin (BSA; sc-2323), goat anti-rabbit IgG-HRP (sc-2004) and antibodies for hypoxia-inducible factor 1-alpha (HIF-1α; sc-10790), Von Willebrand factor (VWF; sc-14014) and glyceraldehyde-3-phosphate dehydrogenase (GAPDH; sc-365062) were from Santa Cruz Biotechnology (Dallas, TX, USA). The HIF-2α antibody (NB-100-122) was from Novus Biologicals (Littleton, CO, USA). NG2 (ab50009) and NDUFS1 (ab169540) antibodies were from Abcam (Cambridge, England). Life Technologies (Carlsbad, CA, USA) was the source for foetal bovine serum (FBS; 10437) Green fluorescent Alexa Fluor 488 isolectin GS-IB4 conjugate (I21411n), dihydroethidium (DHE; D11347) and Prolong Gold Antifade Reagent (P36930). Pierce BCA Protein Assay kit (23225), Restore Western Blot Stripping Buffer (21059), Lerner Aqua-Mount (13800) and enhanced chemiluminescence substrate (32106) were from Thermo Scientific (Waltham, MA, USA). 4′,6-Diamidino-2-phenylindole (DAPI; D9542), goat serum (G9023), Tween 20 (P9416), Triton X-100 (T8532), Oil Red O (O0625) and Haematoxylin Solution, Mayer’s (MHS16) were from Sigma-Aldrich (St. Louis, MO, USA). Protease inhibitor cocktail tablets (11836153001) were from Roche Diagnostics (Basel, Switzerland). Endothelial cell growth medium with supplements (EGM-2 SingleQuot Kit Supplements & Growth Factors, CC-4176) was from Lonza (Basel, Switzerland).

### Animals

Male SIRT3 KO (#012755) and WT (#002448) mice of the same strain (129S1/SvImJ) were obtained from The Jackson Laboratory (Bar Harbor, ME, USA). Mice were housed in the institutional laboratory animal facility (LAF) with free access to food and water. Mice were maintained on a 12 hrs light–12 hrs dark cycle. All procedures conformed to the Institute for Laboratory Animal Research Guide for the Care and Use of Laboratory Animals and were approved by the University of Mississippi Medical Center Animal Care and Use Committee (Protocol ID: 1280). The investigation conforms with the Guide for the Care and Use of Laboratory Animals published by the US National Institutes of Health (NIH Publication No. 85-23, revised 1996).

### High fat DIO model

Male SIRT3 KO or WT mice (8 weeks of age) were fed with normal chow diet (8640 Teklad 22/5 Rodent Diet; Harlan Laboratories (Indianapolis, IN, USA)) or a high-fat (60% kcal) diet (D12492; Research Diets (New Brunswick, NJ, USA)) for 16 weeks to produce a diet-induced obesity model. Mice were housed in the LAF and were given free access to water throughout the study.

### Fasting glucose levels

Blood was obtained from mice by tail snip and blood glucose levels measured using the One Touch SureStep meter. Glucose levels were expressed as mg/dl. Mice were fasted overnight before taking blood. Blood glucose was measured at the start of the study [all mice on normal diet (ND)] and then again after 16 weeks of either ND or HFD.

### Echocardiography

Mice were anaesthetized using a mixture of isoflurane (1.5%) and oxygen (0.5 l/min.). Transthoracic two-dimensional M-mode echocardiography was performed using a Visual Sonics Vevo 770 Imaging System (Toronto, ON, Canada) equipped with a 707B high frequency linear transducer. Short-axis imaging was taken as M-mode acquisition for 30 sec. End-systolic and end-diastolic dimensions, end-systolic and end-diastolic volumes (ESV and EDV), stroke volume, were recorded to calculate the per cent fractional shortening (FS%) and ejection fraction (EF%). Data were analysed using Vevo 770 Analytic Software (FujiFilm VisualSonics Inc., Toronto, Ontario, Canada).

### Immunostaining

Ventricular sections were prepared using a cryostat on microscope slides. Formaldehyde fixed sections were rinsed in PBS and incubated with methanol for 10 min. at −20°C. Slides were washed and then incubated with blocking buffer (5% goat serum/0.3% Triton X-100 in 1× PBS) for 1 hr. Sections were incubated with primary antibody anti-NG2 (1:200) and IB4 (1:50) overnight at 4°C in PBS. Slides were washed and incubated with secondary AlexaFluor488-conjugated anti-rabbit IgG antibody for the NG2 antibody for 1 hr at room temperature in the dark. Sections were incubated with DAPI, washed and cover-slipped with Prolong Gold Antifade Reagent. Images were captured at 20× using an EVOS fl digital inverted fluorescence microscope. Fluorescence was quantified using image-analysis software (Image J, NIH Bethesda, Maryland, USA). Ten randomly chosen images from three sections per heart were analysed.

### Cardiac lipid staining

Cut frozen sections were fixed in formalin, rinsed with 60% isopropanol and stained with freshly prepared Oil Red O working solution for 15 min. After rinsing with isopropanol, nuclei were stained with haematoxylin and sections were cover-slipped using Lerner Aqua-Mount.

### Cell proliferation assay

Microvascular endothelial cells were isolated by cloning from lungs of WT and SIRT3 KO mice and confirmed using VWF and IB4 antibodies. For the proliferation measurement, endothelial cells were plated at the same density (3000 cells per 0.32 cm^2^) and cultured in complete endothelial cell growth media with 10% FBS for 72 hrs. The proliferative capacity of cultured endothelial cells was assayed using a cell proliferation (MTT) kit (11465007001) according to the manufacturer’s instructions (Roche Diagnostic).

### DHE staining

Ventricular sections were prepared using a cryostat on microscope slides. Slides were rinsed in PBS and incubated with 0.5 mM DHE in PBS for 15 min. at room temperature in the dark. Slides were washed and cover-slipped with prolong gold antifade reagent. Relative density of DHE (red) fluorescence was quantified by measuring six random microscopic fields per mouse heart using image-analysis software (Image J, NIH).

### Western blot analysis

Ventricles were collected and sonicated in a RIPA lysis buffer supplemented with protease inhibitor cocktail. Homogenates were centrifuged at 16,200 × g at 4°C for 15 min. Equal amounts of proteins in the supernatants were separated by SDS-PAGE and transferred to polyvinylidene difluoride (PVDF) membranes. Membranes were blocked with 5% w/v powder milk in PBS with 0.1% v/v Tween 20 (PBST) for 1 hr and then incubated with primary antibodies for SIRT3 (1:1000), HIF-1α (1:1000), HIF-2α (1:1000) in 1% w/v BSA in PBST overnight at 4°C. After washing, blots were incubated with horseradish peroxidase conjugated secondary antibody (1:1000) and protein bands visualized using the enhanced chemiluminescence substrate. Blots were stripped using Restore Western Blot Stripping Buffer following the manufacturer’s protocol and reprobed (1:1000) for GAPDH antibody as a loading control. Protein bands were quantified by densitometry with image acquisition and analysis software (TINA 2.0, University of Manchester, United Kingdom).

### Statistical analysis

Unless otherwise noted, data are expressed as mean ± SEM. Statistical significance of differences between two values was determined by a Student’s *t*-test. Two-way anova followed by Tukey’s multiple comparisons test was used for multiple comparisons and to assess interaction between SIRT3 KO and HFD, the significance of which is denoted as *P*_I_. The significance of the effect of diet or SIRT3 loss is denoted *P*_D_ and *P*_S_, respectively. *P* ≤ 0.05 was considered statistically significant. Non-significance (*P* > 0.05) is denoted as *ns*.

## Results

### High-fat diet reduces SIRT3 expression in mouse heart and leads to cardiac hypertrophy

**Figure 1:**
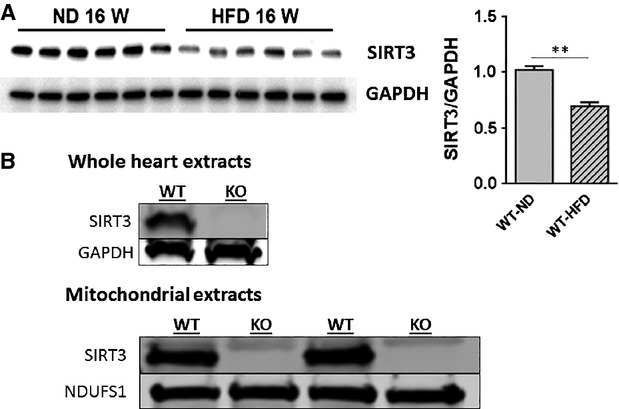
HFD decreases SIRT3 expression in the heart. (A) WT mice were fed ND or HFD for 16 weeks. Hearts were extracted and Western blot analysis was performed on ventricular lysates. The blot was probed for SIRT3 expression. The blot was stripped and reprobed for GAPDH as a loading control. Bar graph shows quantification of expression. Values are mean ± SEM, *n* = 6 mice per group. **P < 0.01. (B) Confirmation that KO mice do not express SIRT3. Protein extracts from the ventricles and isolated mitochondria of WT and SIRT3 KO mouse hearts were probed by Western analysis for SIRT3 (28 kD). For heart extracts, GAPDH (37 kD) was probed for to demonstrate equal protein loading; for mitochondria extracts, Complex I subunit NDUFS1 was used as the loading control (∼75 kD) for mitochondrial samples. Results are from 3 WT and 3 KO mice.

We first examined SIRT3 expression in the hearts of HFD mice. Feeding mice with HFD for 16 weeks led to a significant reduction in SIRT3 expression in the heart. SIRT3 levels were decreased by 32% in the HFD group compared to the ND group (Fig.1A). We therefore proceeded to compare the consequences of HFD on the heart of WT to SIRT3 KO mice. As shown in Figure1B, we confirmed that SIRT3 KO mice do not express this protein in heart whole tissue or mitochondria extracts.

High-fat diet caused comparable increases in heart weight and cardiac hypertrophy, as indexed by the heart weight to tibia length ratio, in both WT and SIRT3 KO mice (Table1). For WT mice, there was an increase in both left ventricular end-diastolic and end-systolic dimensions (LVDD and LVSD), and the calculated EDV and ESV. This finding along with the increase in heart weight/tibia length ratio is consistent with an eccentric pattern of LV hypertrophy. Although hearts of SIRT3 KO mice on ND were not hypertrophied, LVDD, LVSD, EDV and ESV, were greater in the SIRT3 KO mice fed ND than the WT mice on the same diet, suggesting fundamental differences in heart structure between WT and SIRT3 KO mice. SIRT3 influenced LVDD and LVSD, as well as EDV and ESV; HFD and absence of SIRT3 each increased these parameters and there was no further increase in mice when these factors were combined (*P*_I_ ≤ 0.05); in fact, there appears to be a more complex LVDD and EDV response when SIRT3 KO mice are fed a HFD. This interpretation is based on a comparison of the LVDD and EDV values for WT and SIRT3 KO mice fed HFD. Relative to the values seen in WT mice on ND, there was a tendency for the increase in these values to be less in SIRT3 KO mice fed a HFD than WT mice fed a HFD (Table1). There were no differences in heart rate because of loss of SIRT3 or diet.

**Table 1:** Biometric and echocardiographic data of WT and SIRT3 KO mice fed ND and HFD for 16 weeks

	WT ND	WT HFD	SIRT3 KO ND	SIRT3 KO HFD
Heart weight (g)	0.132 ± 0.004 (8)	0.164 ± 0.004 (8)*,†	0.123 ± 0.001 (7)	0.151 ± 0.006 (7)†,‡
*P*_I_ = *ns*; *P*_D_ ≤ 0.01; *P*_S_ ≤ 0.05				
HW/tibia length (g/cm)	0.0774 ± 0.0023 (8)	0.0922 ± 0.0025 (8)*,†	0.0733 ± 0.0008 (7)	0.0953 ± 0.0040 (7)*,†
*P*_I_ = *ns*; *P*_D_ ≤ 0.01; *P*_S_ = *ns*				
LVDD (mm)	3.48 ± 0.16 (7)	4.20 ± 0.06 (7)*	4.02 ± 0.12 (8)‡	3.77 ± 0.11 (9)
*P*_I_ ≤ 0.01; *P*_D_, *P*_S_ = *ns*				
LVSD (mm)	2.31 ± 0.11 (7)	2.98 ± 0.05 (7)*	2.95 ± 0.15 (8)*	3.08 ± 0.07 (9)*
*P*_I_ ≤ 0.05; *P*_D_, *P*_S_ ≤ 0.01				
EDV (μl)	51.18 ± 5.62 (7)	78.70 ± 2.57(7)*	71.55 ± 4.94 (8)‡	63.50 ± 3.09 (9)
*P*_I_, *P*_D_ ≤ 0.01; *P*_S_ = *ns*				
ESV (μl)	18.85 ± 2.15 (7)	34.62 ± 1.32 (7)*	34.97 ± 4.29 (8)*	37.68 ± 2.16 (9)*
*P*_I_ ≤ 0.05; *P*_D_, *P*_S_ ≤ 0.01				
Heart rate (beats/min.)	405 ± 7 (8)	410 ± 8 (8)	415 ± 10 (10)	412 ± 10 (10)
*P*_I_, *P*_D,_ *P*_S_ = *ns*				

* *P* ≤ 0.01 *versus* WT ND.

† *P* ≤ 0.01 *versus* SIRT3 KO ND.

‡ *P* ≤ 0.05 *versus* WT ND.

EDV: end-diastolic volume; ESV: end-systolic volume; HW: heart weight; LVDD: left ventricular end-diastolic dimension; LVDS: left ventricular end-systolic dimension; HFD: high-fat diet; ND: normal diet.

### Loss of SIRT3 exacerbates HFD-induced ROS formation

**Figure 2:**
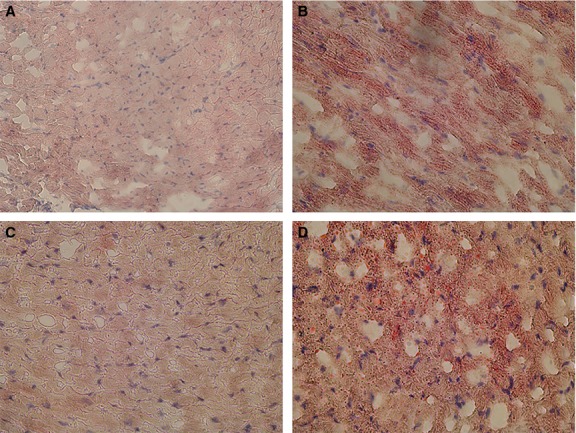
Accumulation of lipids in hearts of mice fed HFD. Ventricular slices were stained for lipids using Oil Red O. Representative images are shown for hearts from WT mice fed (A) ND, or (B) HFD and SIRT3 KO mice fed a (C) ND or (D) HFD.

**Figure 3:**
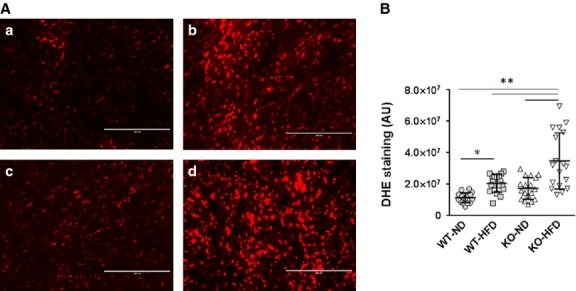
Detection of increased ROS levels in the hearts of WT and SIRT3 KO mice on HFD. Hearts were extracted from WT and KO mice fed ND and HFD for 16 weeks. Ventricular sections were stained with dihydroethidium (DHE), which forms a red colour when bound to ROS-damaged DNA. (A) Representative images of DHE staining of hearts from WT-ND, WT-HFD, KO-ND and KO-HFD mice (a, b, c and d respectively) are shown (*n* = 3 mice per group); bar = 200 μm. (B) DHE staining was quantified using six sections selected randomly from each heart. Black bars show mean ± SD. *P*_I_ = *ns*, *P*_D_ and *P*_S_ ≤ 0.01; *P ≤ 0.05; **P ≤ 0.01.

Feeding mice a HFD for 16 weeks lead to an accumulation of lipids in the hearts of both WT and SIRT3 KO mice (Fig.2). In addition, hearts from mice fed with HFD for 16 weeks exhibited a significant increase in DHE staining, indicating increased ROS levels, compared to mice fed with ND (Fig.3A and B). There was a trend towards increased ROS levels in hearts of SIRT3 KO mice on ND compared to WT mice on ND, but this did not reach significance. However, SIRT3 KO mice fed HFD exhibited a significant increase in DHE staining in the heart (Fig.3A and B). No interaction was observed between HFD and SIRT3 loss on ROS formation in the heart; however, the consequences of HFD on oxidative stress in the heart were enhanced by SIRT3 loss.

### High-fat diet-induced obesity but not a diabetic state

**Figure 4:**
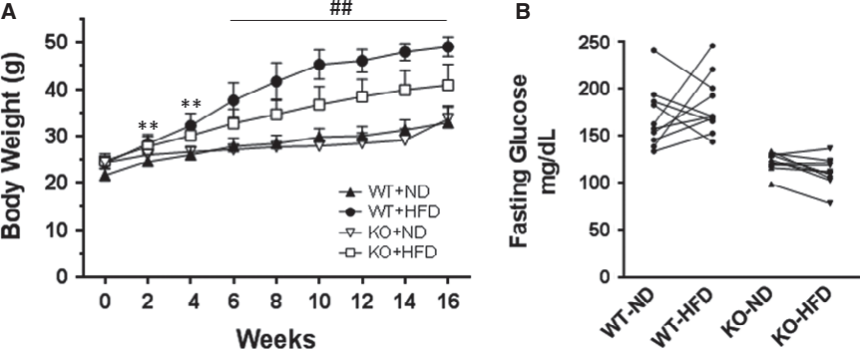
Effect of HFD on bws and fasting blood glucose levels in mice. WT and SIRT3 KO mice were fed a HFD and ND for 16 weeks. (A) Body weight curves over 16 weeks are shown. Values are shown as mean ± SD, *n* = 10 mice per group. *P*_I_ ≤ 0.01; **P ≤ 0.01 WT-ND *versus* WT-HFD; ^##^*P* ≤ 0.01 WT-HFD *versus* WT-ND, KO-HFD *versus* KO-ND, and WT-HFD *versus* KO-HFD. (B) Blood glucose measurements were made in WT and SIRT3 KO mice fasted for 24 hrs. Measurements were made in individual mice on ND and following 16 weeks of HFD (10 mice per group).

A diet of 60% fat caused a notable increase in bw in WT mice over the course of 16 weeks (Fig.4A). Body weight increased as well in SIRT3 KO mice on HFD, although to a lesser degree (*P*_I_ ≤ 0.01). In contrast, others reported that feeding SIRT3 KO mice a so-called Western diet of high fat (42% kcal) and high carbohydrate (42.7% kcal), more than 2× the carbohydrate content of our diet, accelerated obesity and led to development of the metabolic syndrome 9. Mitochondrial dysfunction at basal conditions in SIRT3 KO may explain why SIRT3 KO mice gained less bw with HFD (mitochondrial stress) than WT mice as fatty acid oxidation would be more prominent in our study. No difference was seen over the 16 weeks in bw between WT and SIRT3 KO mice fed ND. Neither HFD nor SIRT3 KO induced diabetes. Fasting glucose levels were not increased in either WT or SIRT3 KO mice on HFD compared to ND (Fig.4B). Others have noted that the ability of HFD to induce diabetes in mice is strain-dependent 10. Of note, SIRT3 KO mice exhibited significantly lower fasting blood glucose levels than WT mice on the same diet (*P* ≤ 0.01); lower blood glucose levels may explain as well why SIRT3 KO mice gained less weight with HFD than WT mice.

### SIRT3 deficiency promotes high-fat diet-induced cardiac dysfunction

**Figure 5:**
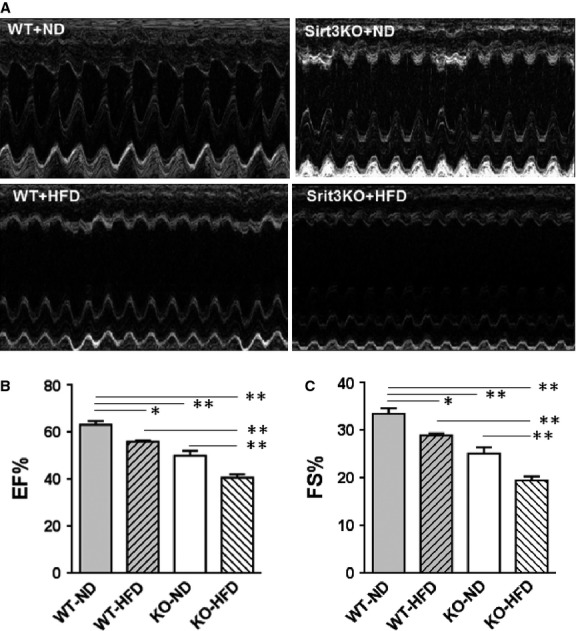
Assessment of cardiac function by echocardiography. (A) Representative M-mode tracings are shown. (B) Ejection fraction and (C) fractional shortening were determined. Values are means ± SEM, *n* = 10. *P*_I_ = *ns*, *P*_D_ and *P*_S_ ≤ 0.01; *P ≤ 0.05; **P ≤ 0.01.

Mice fed a HFD exhibited a modest decline in cardiac function. As seen in Figure5, EF and FS were significantly decreased in WT mice fed HFD compared to mice fed ND for 16 weeks. Knocking out SIRT3 under ND also decreased cardiac function compared to WT mice fed ND. High-fat diet treatment further reduced cardiac performance in SIRT3 KO mice (Fig.5) to an extent that on average was greater than for WT mice; however, no interaction between HFD and SIRT3 loss was observed.

### High-fat diet and SIRT3 loss reduce HIF-1α and HIF-2α expression in the heart

**Figure 6:**
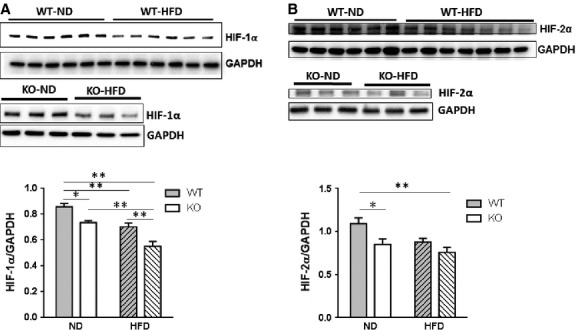
HIF-1α and HIF-2α are reduced by HFD and SIRT3 loss in the heart. WT and SIRT3 KO mice were fed ND or HFD for 16 weeks. Hearts were extracted and Western blot analysis was performed on ventricular lysates. (A) Representative blot of HIF-1α. Blot was stripped and reprobed for GAPDH as a loading control. Bar graph shows mean ± SEM for *n* = 6. *P*_I_ = *ns*, *P*_D_ and *P*_S_ ≤ 0.01; *P ≤ 0.05; **P ≤ 0.01. (B) Representative blots of HIF-2α and GAPDH. Values are mean ± SEM (*n* = 6 ND and 5–7 HFD). *P*_I_ = *ns*, *P*_D_ ≤ 0.05, *P*_S_ ≤ 0.01; *P ≤ 0.05; **P ≤ 0.01.

We previously reported that impaired HIF signalling is associated with reduced myocardial capillary density in obese diabetic mouse model 11. Hence, we examined HIF levels in diet-induced obesity model. As seen from Fig.6A and B, cardiac HIF-1α and HIF-2α levels were reduced in mice fed HFD compared to mice fed ND for 16 weeks. Hypoxia-inducible factor-1α and -2α levels were also significantly reduced in hearts of SIRT3 KO mice compared to WT mice (Fig.6A and B). Hypoxia-inducible factor-1α levels were further decreased in SIRT3 KO-DIO mice compared to SIRT3 KO mice (Fig.6A and B). Levels of HIF-2α tended to be further reduced by HFD in SIRT3 KO hearts, but this did not reach significance. No interaction between HFD and SIRT3 loss on HIF-1α and -2α levels was demonstrated.

### High-fat diet and SIRT3 KO cause capillary rarefaction and loss of pericytes

**Figure 7:**
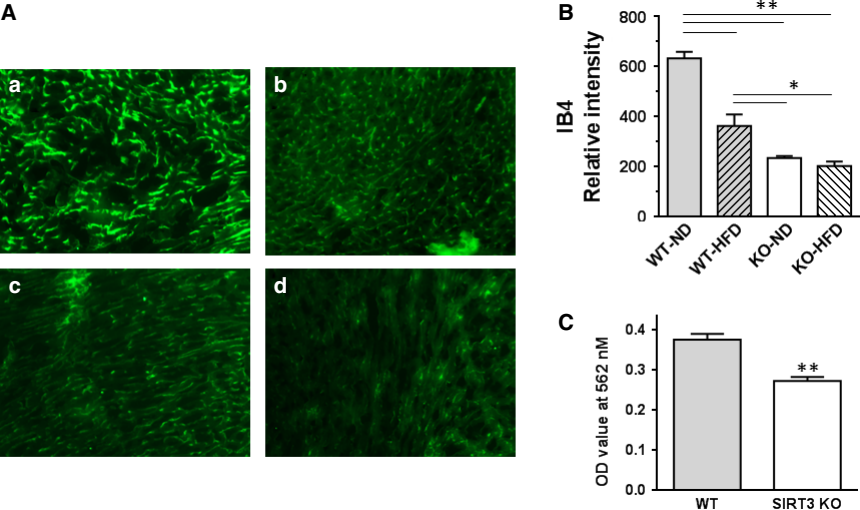
HFD and SIRT3 loss reduce capillary density in the heart. Hearts were extracted from WT and KO mice fed ND or HFD for 16 weeks. Ventricular sections were prepared and stained with green fluorescent isolectin B4 (IB4) as an endothelial cell marker. (A) Representative images of mouse hearts from WT-ND, WT-HFD, KO-ND and KO-HFD (a, b, c and d respectively) are shown. (B) IB4 staining was quantified by Image J and normalized to DAPI staining of nuclei (relative intensity). Values are mean ± SEM, *n* = 3 mice per group. *P*_I_, *P*_D_ and *P*_S_ ≤ 0.01; *P ≤ 0.05; **P ≤ 0.01. (C) Cell proliferation assay. The proliferation of microvascular endothelial cells from WT and SIRT3 KO lungs was assessed using an MTT assay. Values are mean ± SEM, *n* = 5 independent observations; **P ≤ 0.01.

We next explored whether impairment of HIF-α signalling affected the vasculature of the heart as cardiac dysfunction is associated with decreases in capillary density and coronary blood flow. Wild-type mice fed with HFD showed a marked decrease in capillary density compared to WT mice fed ND (Fig.7A and B). Intriguingly, greater loss of capillary density was observed in SIRT3 KO mice, which was not further enhanced by HFD (*P*_I_ ≤ 0.01). This finding indicates that SIRT3 loss contributes to the decrease in capillaries seen with HFD. As Figure7C shows, we observed that deletion of SIRT3 markedly reduced proliferation of endothelial cells, which likely contributed to the reduction in capillaries with HFD.

**Figure 8:**
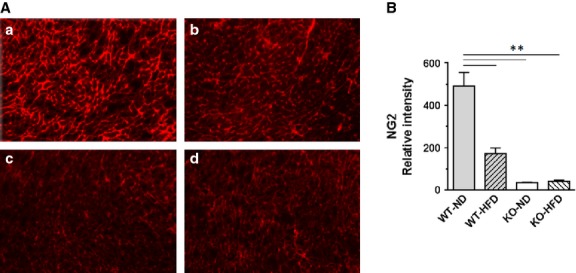
HFD and SIRT3 KO reduce pericyte density in the heart. Hearts were extracted from WT and KO mice fed ND and HFD for 16 weeks. Ventricular sections were stained with an antibody to NG2 to detect pericytes. Binding of NG2 was detected using a secondary antibody that fluoresces red. (A) Representative images of NG2 staining of mouse hearts from WT-ND, WT-HFD, KO-ND and KO-HFD (a, b, c and d respectively) are shown. (B) NG2 staining was quantified using Image J and normalized to DAPI staining. Values are mean ± SEM, *n* = 3 mice per group. *P*_I_, *P*_D_ and *P*_S_ ≤ 0.01; **P ≤ 0.01.

Loss of pericytes has been shown to promote capillary rarefaction in pancreatic islets and skeletal muscle, thus leading to or exacerbating glucose intolerance 12,13. As Figure8A and B show, HFD also resulted in a dramatic loss of pericytes in WT mice. The loss of pericytes was even greater with SIRT3 KO mice and no further loss of pericytes was observed in SIRT3 KO-DIO mice (*P*_I_ ≤ 0.01), suggesting a critical contribution of reduced SIRT3 in the actions of HFD on pericytes.

## Discussion

In our study, we report the following novel observations: (*i*) HFD reduced SIRT3 levels in the heart and caused cardiac systolic dysfunction; (*ii*) either HFD or SIRT3 KO increased ROS levels, which was associated with impaired HIF signalling and reduced myocardial capillary density and (*iii*) combined HFD and SIRT3 KO synergistically elevated ROS levels and caused greater cardiac dysfunction. However, no interaction (*i.e*. antagonism or synergism) was observed between HFD and SIRT3 loss on cardiac ROS production, systolic function or hypertrophy. This observation indicates that HFD acted also by means other than SIRT3 repression to adversely affect heart function and structure. Notably, both HFD and SIRT3 loss produced a marked reduction in capillary and pericyte density that was not further increased by their combination, suggesting a common underlying mechanism.

Most studies have reported that HFD induces cardiac dysfunction, but the mechanism by which this occurs is unclear. In this study, we hypothesized that HFD reduces SIRT3 expression, which would lead to increased ROS production and cardiac dysfunction. Loss of SIRT3 was correlated with increased ROS levels and mitochondrial dysfunction in the heart 14,15, while overexpression of SIRT3 was shown to protect cardiac myocytes from oxidative stress and apoptosis 16–18. We tested our hypothesis by using SIRT3 KO mice and found that loss of SIRT3 showed similar cardiac phenotype as HFD with increased ROS production and modest cardiac dysfunction. We therefore further tested our notion that impaired mitochondrial function exacerbates diet-induced heart failure by feeding SIRT3 KO mice a HFD, creating a model of metabolic/mitochondrial stress. This resulted in even higher ROS levels and a greater decline in cardiac function. Our data indicate that HFD only induces modest cardiac dysfunction and additional factors such as mitochondrial dysfunction are required to promote heart failure in obesity.

While we were doing our study, another group reported that HFD switches cardiac metabolism from glucose to fatty acid oxidation and showed reduced SIRT3 expression as the underlying cause 19. The basis for the decrease in SIRT3 levels with HFD is not defined; however, a recent study showed that ROS-mediated NF-κB activation down-regulates SIRT3 levels in cardiomyoblasts 20. Thus, decreased SIRT3 could promote further loss of SIRT3 *via* a positive feedback mechanism involving ROS.

SIRT3, a NAD^+^ dependent deacetylase, belongs to class III histone deacetylases. SIRT3 is a mitochondrial protein whose increased expression has been shown to be associated with longevity of humans 21,22. Older individuals have about a 40% reduction in SIRT3, and the health benefits of older patients were accompanied by elevated levels of SIRT3 23. Loss of SIRT3 has been related to cardiac hypertrophy in ageing 14,24. Thus, diet-induced obesity SIRT3 KO (SIRT3 KO-DIO) mice may be useful as a novel model to study HFD-induced heart failure in ageing.

In our study, HFD and SIRT3 KO mice showed increased levels of ROS in the heart (Fig.3). In addition to a direct damaging effect on the heart, increased ROS may also impair HIF signalling in the heart. HIFs are transcription factors that are activated under hypoxic condition. Two isoforms, HIF-1α and HIF-2α, have similar structure and function (bind to the same hypoxia responsive element). Although they vary in their tissue specific expression pattern, both are expressed in the heart. We observed that HFD or SIRT3 KO reduced HIF-1α and -2α signalling in the heart (Fig.6). Increased ROS levels and α-ketoglutarate because of reduced activity of Krebs cycle, both associated with SIRT3 deficiency, have been linked to reduced HIF signalling activation 25. However, our evidence indicates that HFD-reduced HIF signalling in the heart is largely independent of SIRT3 loss (Fig.6).

High-fat diet or SIRT3 KO reduced density of endothelial cells and pericytes in the heart (Figs7 and 8). Reduction in HIF-1α and -2α levels may explain in part the myocardial capillary loss in mice fed with HFD, as HIF signalling is important for angiogenesis and endothelial cell survival 26. Impaired HIF signalling would down-regulate angiogenesis, ultimately resulting in reduced capillary density in the heart. Recently, histological analysis of non-ischaemic myocardium from patients undergoing coronary artery bypass surgery showed that obesity was associated with lower coronary microvascular density 27. A previous study from our lab showed that overexpression of angiopoietin 1 improved capillary density in ischaemia-induced obese *db/db* mouse hearts *via* up-regulating HIF-1α expression 11. In subsequent studies, we demonstrated that overexpression of apelin improved myocardial capillary density and alleviated diabetic cardiomyopathy *via* SIRT3 up-regulation in *db/db* mice 7.

Overall, our results indicate that HFD induces an inappropriate response in the heart as there is a reduction in pro-angiogenic HIF signalling in the face of a loss in capillary density. But ROS and loss of HIF signalling alone cannot explain impaired angiogenesis seen with HFD. Our evidence indicates that HFD impairs angiogenesis *via* repression of SIRT3 (Fig.7A and B). Impaired microvascular endothelial cell proliferation (Fig.7C) and loss of pericytes (Fig.8) were likely contributing factors.

A correlation between reduced vascular density and cardiac dysfunction has been previously reported. For instance, a recent study showed reduced capillary density and cardiac dysfunction in mice subjected to transverse aortic constriction 28. However, while our study results suggest that loss of capillary density contributed to cardiac dysfunction, other factors such as ROS production likely contributed as well. This is because HFD in SIRT3 KO mice did not further reduce capillary density, but cardiac function was made worse.

Our data from the SIRT3 KO mouse indicate the relationship between (total knockout of) SIRT3 and cardiac dysfunction is complex. For example, evidence for the ROS component of the pathway suggests there is a subset of SIRT3 KO mice that developed exacerbated increases in ROS levels, and further reductions in HIF protein levels. Increased ROS and impaired HIF signalling resulting from SIRT3 loss may have contributed to impaired vascularization; however, cell-type specific responses are likely involved as well, including impaired endothelial cell proliferation and loss of pericytes. Nor does there seem to be a straightforward link between impaired contractile function with SIRT3 KO and reduced vascularization. Here also cell-type specific factors in cardiac myocytes, such as the status of energy stores and contractile proteins, must be considered. Nevertheless, our data highlight the role of a reduction in SIRT3 as a contributing factor to the pathological effects of HFD on the heart.

A limitation of our study is that we did not assess the metabolic components that may have contributed to the decline in contractile function along with increased oxidative stress. In this regard, SIRT3 KO mice did gain slightly less weight on HFD than WT mice (Fig.4A). Those experiments exceed the focus of this study and are currently being undertaken. It seems unlikely though that HFD by itself would compromise the ability of the heart to generate sufficient ATP to sustain contractile function. An additional consideration is that the SIRT3 KO is a whole body knockout. The finding that fasting glucose is lower in the KO *versus* WT mice suggests cells outside the myocardium are likely playing a role in the KO phenotype and possibly the response to HFD. Another limitation is that the data also do not eliminate the possibility for a role of cytoplasmic or nuclear SIRT3.

In conclusion, our findings suggest that HFD and SIRT3 loss compromise heart function and increase production of ROS levels in the heart. However, although HFD reduced SIRT3 levels in the heart, the adverse effects of HFD on cardiac remodelling and function cannot be attributed solely to SIRT3 loss. Our results also show that applying metabolic stress by feeding HFD to SIRT3 KO mice further impairs cardiac function. This is likely because of enhanced ROS generation resulting from increased mitochondrial oxidation with HFD, exacerbated by the absence of SIRT3. Our study thus identifies SIRT3 as a potential therapeutic target for preventing obesity-induced cardiac dysfunction.
